# Study of disease phenotype and its association with prognosis of paediatric inflammatory bowel disease in China

**DOI:** 10.1186/s12887-018-1212-x

**Published:** 2018-07-12

**Authors:** Xin-Qiong Wang, Yuan Xiao, Xu Xu, Yi Yu, Cheng-Yan Shan, Yan Guo, Ling Gong, Tong Zhou, Shen-Shen Gao, Yao-Zong Yuan, Xiao-Jin Wang, Chun-Di Xu

**Affiliations:** 1 Department of Paediatrics, Ruijin Hospital, Shanghai Jiao Tong University, School of Medicine, No. 197, Rui Jin Er Road, Shanghai, 200025 China; 2 Department of Paediatrics, Ruijin Hospital North, Shanghai Jiao Tong University, School of Medicine, Shanghai, 201821 China; 3 Department of Gastroenterology, Ruijin Hospital, Shanghai Jiao Tong University, School of Medicine, Shanghai, 200025 China; 4 Department of Biostatistics, Shanghai Jiao Tong University, School of Medicine, Shanghai, 200025 China

**Keywords:** Inflammatory bowel disease, Children, Infantile or toddler onset IBD, Paris classification, Prognosis

## Abstract

**Background:**

To investigate the unique features of inflammatory bowel disease (IBD) in children, we wanted to identify whether there might be a strong correlation between the disease phenotype and its prognosis at various ages in paediatric patients.

**Methods:**

We collected data from patients diagnosed with IBD (ulcerative colitis (UC) or Crohn’s disease (CD)) from 2002 to 2016. The diagnosis was made according to the Porto criteria and Paris Classification. Patient characteristics, clinical manifestations and treatments were collected. Risk factors for surgery, mortality and relapse were analysed by Cox proportional hazard models.

**Results:**

Of the 143 patients, 113 had CD, and 30 had UC; there were 89 males and 54 females with a median age of 9 years (y). Thirteen patients in the 0–2 y group were identified as having mutations in IL-10 receptor A, and this mutation was significantly more common in this age group than in 3–9 and 10–16 y patients. The risk factor for surgery was the B3 phenotype; risk factors for death were age 0–2 y and B3 phenotype; 0–2 y, B3 phenotype and steroid dependency were risk factors for early relapse.

**Conclusions:**

Clinical manifestations of the onset of IBD in infants and toddlers were extensive and aggressive and were closely associated with early relapse and death. It is of particular interest that some of these patients developed IBD due to monogenic disorders; thus, introduction of genetic testing is essential for these patients.

## Background

Inflammatory bowel disease (IBD) includes Crohn’s disease (CD) and ulcerative colitis (UC); IBD-unclassified (IBD-U) is a group of chronic gastrointestinal inflammatory diseases. Approximately 25% of patients manifest with the disease in childhood or adolescence [1]. Our previous studies have indicated that the morbidity of paediatric IBD has been rapidly increasing in China over the past three decades [2]. Studies have also shown that paediatric IBD manifests as an extensive and aggressive disease [3, 4]. However, further study suggested that the clinical manifestations and prognosis varied greatly in patients with various onset ages [5]. In our study, the paediatric Paris classification released in 2013 was used, as it is valuable for paediatric IBD studies [6]. Using the Paris classification, we further sub-classified IBD patients into two groups, A1a (0–10 y) and A1b (10–17 y). We also focused on the children with very early onset IBD (VEO-IBD), including infantile and toddler onset IBD, as infantile IBD might be partially linked to monogenic diseases such as defects in IL-10 or its receptors, Wiskott-Aldrich Syndrome, XIAP deficiency, leukocyte adhesion deficiencies, CD40L deficiency, IPEX syndrome and several others [7–9]. The clinical manifestations and phenotypes in this group of patients were different from those of patients in other age groups. However, it remains controversial as to whether these patients with monogenic diseases phenotype should be classified as having IBD [9, 10].

Because the clinical manifestations and phenotypes vary in IBD children, the prognosis of IBD is remarkably different in patients of different ages, and there is a lack of long-term follow-up studies on the natural course of the disease. In the current study, the natural course of disease was recorded with long-term follow-up to define the features and progression of paediatric IBD in China.

## Methods

Medical records were retrospectively selected from the Department of Paediatrics, Ruijin hospital and North Ruijin Hospital; patients were diagnosed as having UC, CD, or IBD-U from January 2002 to September 2016. As a paediatric IBD centre, patients suspected of having IBD were recorded and followed up. The diagnosis was confirmed by at least three gastrointestinal (GI) paediatricians after complete physical examination, endoscopy, pathological examination, and radiological imaging determinations. The diagnosis was made according to the Porto criteria and Paris classification [6, 11, 12]. Complex patients with unclear diagnosis were re-evaluated by a multi-disciplinary team (MDT) of IBD professionals, consisting of GI paediatricians, radiologists, nutritionists, surgeons, nurses and adult gastroenterologists. Once the diagnosis was confirmed, the patients were followed up at the outpatient department regularly, and some patients were admitted to hospital for further treatment if necessary. A well-trained administrative staff was assigned to collect, document and store all the data.

There were 200 patients primarily reviewed. Fourteen diagnosed as having IBD-U could not be precisely classified until final follow-up and were therefore excluded from this study. Thirty-two patients with a follow-up period of less than 6 months were also excluded; however, the 11 patients who died within 6 months after diagnosis were included. Another 11 patients with incomplete medical records or without reports of endoscopy or imaging examination were excluded as well. Finally, there were 143 patients included in this study. All were less than 17 years old at the time of diagnosis. The patients were classified into three groups according to their age at diagnosis: 0–2, 3–9 and 10–16 years old groups. Clinical information and laboratory tests were collected at diagnosis and at each follow-up.

### Genetic workup

Twenty-four patients with onset before 3 years old had a genetic test (20 in the 0–2 y group and 4 in the 3–10 y group). Thirteen patients were involved in a previously published study that tested for 10 genes [13]; three of these had subsequent whole exome sequencing (WES). The other 10 patients with VEO-IBD were further suggested to undertake genetic tests, comprising of more than 50 genes [14, 15] that were closely related to VEO-IBD (medical exome sequencing). In addition, parents were verified by Sanger sequencing if any positive finding was detected in the IBD children. One 5-year-old patient was confirmed as having glycogen storage disease (GSD) Ib prior to IBD diagnosis.

### Disease activity index and definition of other evaluation indexes

Disease activity was assessed by the Paediatric Crohn’s Disease Activity Index (PCDAI) for patients with CD [16], and the Paediatric Ulcerative Colitis Activity Index (PUCAI) for patients with UC [17]. Patients with PUCAI ≥65 were classified as having severe disease according to the Paris classification. In terms of disease progression, the duration between diagnosis and first relapse after clinical remission was recorded for each patient. A patient was defined as being in clinical remission if the disease activity index was < 10 after induction therapy until the last follow-up, whereas a patient was defined as not in remission or relapse if the disease activity index was ≥10 with symptoms after induction therapy. Steroid dependency was defined as a patient receiving more than 10 mg/d prednisolone for more than 3 months or clinical relapses were seen within 3 months of tapering steroids. Patients starting biological agents early after diagnosis were regarded as receiving “top-down” treatment.

### Growth and developmental index

Height and weight were two important factors that were routinely recorded at primary diagnosis as well as at subsequent follow-up examinations in order to monitor physical growth and development; the index score was calculated by the Z-scoring method based on the national survey on growth of children under 7 years of age in nine cities of China in 2005 [18]. Z < − 2 for weight at primary diagnosis and follow-up examination was recorded, and growth impairment (G1) was defined by the criteria of the Paris classification [6].

### Statistical analysis

Discrete variables are expressed as numbers and percentages. Quantitative variables of normal distribution were expressed as the mean ± standard deviation (SD). Data was analysed by t-test and chi-squared test to compare categorical data between different age groups. Quantitative variables of skewed distribution were expressed as median and interquartile range and compared by Kruskal-Wallis Wilcoxon rank sum test. Differences were considered statistically significant at *P* < 0.05. Risk factors for surgery, death, and relapse were measured using Cox proportional hazard models for clustered data. Age group, gender, location and behaviour, nutrition status and treatment were included, and factors with a *P* < 0.05 in univariate analysis were included in multivariate marginal Cox proportional hazard regression to create the adjusted model and their corresponding hazard ratio (HR) and 95% CI (confidence interval). Cumulative probabilities of death, surgery and relapse rate in various age groups were calculated using the Kaplan–Meier method. SPSS 19 (Chicago, IL) was used for statistical analyses. GraphPad Prism 5 was used for Kaplan-Meier pictures.

## Results

### Patient characteristics

A total of 143 IBD patients (113 CD and 30 UC) aged under 17 years old were followed for a total of 404.08 person-years with a median follow-up duration of 26 months (range, 0–175 months). The median age at diagnosis was 9 years old. The youngest patient was 2 months old and the oldest was 16 years old. A total of 119 (83.2%) patients were from other provinces (19 provinces) and 64 (44.8%) patients were referred from other hospitals. There were 14 patients confirmed to have monogenic diseases and 13 of these had IL-10 receptor A (IL-10 RA) defects; they were all younger than 3 years old at the time of diagnosis. The mutations of four patients were homozygous, while the others were compound heterozygotes. Eight mutation sites were found; according to the guidelines for the interpretation of sequence variants by the ACMG (American College of Medical Genetics) [19], six sites were classified as pathogenic (p.R101W, p.T179 T, p.R117H, p.G141R, p.W424X and p.R165X) and two were classified into likely pathogenic (p.V100G and p.Y64C). One five-year-old patient was diagnosed as having GSD Ib (compound heterozygote of SLC37A4, two pathogenic sites) prior to IBD diagnosis. Family history of IBD was identified in six patients, and four of these had IL-10 RA defects. No consanguinity of parents was found in any patient.

### Clinical manifestations

Table 1 shows the clinical manifestations of all groups of patients with three different ages. The findings indicated that clinical manifestations varied according to age. The percentage of diarrhoea and blood in stool were relatively high in the 0–2 y group. Other systemic complications, including fever (73.5%), anaemia (76.5%), and growth impairment (55.9%) were also commonly found in this group.

**Table 1:** Patient characteristics and clinical manifestations at diagnosis of different age groups

Characteristics	0–2 y	3–9 y	10–16 y	*P* value
Number (%)	34 (23.8)	46 (32.2)	63 (44.0)	
Male sex, n (%)	24 (70.6)	28 (60.9)	37 (58.7)	0.50
Diagnosis CD, n (%)	30 (88.2)	31 (67.4)	52 (82.5)	0.05
Genetics disease, n (%)	13 (38.2)	1 (2.2)	0 (0.0)	< 0.01
Family history of IBD, n (%)	5 (14.7)	1 (2.2)	0 (0.0)	< 0.01
Median time from symptom onset to diagnosis, mo (IQR)	5 (9.3)	4 (10.3)	4.5 (8.0)	0.41
Median duration of follow-up period, mo (IQR)	9 (22.3)	38 (45.0)	31 (46.0)	< 0.01
Symptoms n (%)				
Abdominal pain	N/A^a^	35 (76.1)	49 (77.8)	0.84
Diarrhoea	32 (94.1)	36 (78.3)	38 (60.3)	< 0.01
Blood in stool	26 (76.5)	30 (65.2)	30 (47.6)	0.02
Oral ulcer	7 (20.6)	5 (10.9)	13 (20.6)	0.36
Fever	25 (73.5)	21 (45.7)	21 (33.3)	< 0.01
Anaemia	26 (76.5)	30 (65.2)	41 (65.1)	0.47
weight < -2SD	16 (47.1)	8 (17.4)	10 (15.9)	< 0.01
Growth impairment	19 (55.9)	18 (39.1)	13 (20.6)	< 0.01
limitation of activities	17 (50)	13 (28.3)	14 (22.2)	< 0.01
Joints	0 (0)	5 (10.9)	1 (1.6)	0.02
Skin	2 (5.9)	1 (2.2)	1 (1.6)	0.50

^a^As the presence of abdominal pain in younger children is very difficult to identify, we did not calculate the numbers of 0–2 y group and only compared the other two groups

### Classification and location of the disease

Paris classification of UC and CD at diagnosis is shown in Table 2. The location of CD varied according to age. The lesions were located mainly in L2 (colonic) in the 0–2 y group; lesions were located mainly in L3 (ileocolonic) in groups 3–9 y and 10–16 y. Thirty-five of 113 (31.0%) CD patients showed upper GI tract lesions based on macroscopic appearance of mucosal ulceration or bowel wall thickening on radiography, and there was one 16-year-old patient with an upper GI lesion only, without colonic or ileocaecal lesions. The behaviour of disease also differed amongst groups. The 0–2 y group showed such lesions as B2 or B2B3 at relatively high percentages. The disease activity was higher in the 0–2 y group than in others at the time of diagnosis.

**Table 2:** Paris phenotype and disease activity at diagnosis of different age groups

	0–2 y	3–9 y	10–16 y	*P* value
UC disease extent, n (%)				
E1 proctitis	0 (0)	2 (13.3)	1 (9.1)	0.80
E2 left-sided colitis	1 (25.0)	5 (33.4)	3 (27.3)	
E3 extensive colitis	2 (50.0)	2 (13.3)	2 (18.2)	
E4 pancolitis	1 (25.0)	6 (40.0)	5 (45.4)	
severe (PUCAI≥65)	0 (0)	3 (20.0)	3 (27.3)	0.35
CD disease location, n (%)				
L1: terminal ileum	2 (6.7)	4 (12.9)	12 (23.1)	< 0.01
L2: colonic	20 (66.6)	5 (16.1)	6 (11.5)	
L3: ileocolonic	8 (26.7)	22 (71.0)	33 (63.5)	
Upper gastrointestinal, n (%)				
L4a + b:	0	0	7 (13.5)	0.03
L4a	4 (13.3)	8 (25.8)	13 (25.0)	
L4b	1 (3.3)	1 (3.2)	1 (1.9)	
CD disease behaviour, n (%)				
B1: non-stricturing, non-penetrating	10 (33.3)	15 (48.4)	23 (44.2)	0.04
B2: stricturing	13 (43.3)	8 (25.8)	27 (51.9)	
B3: penetrating	5 (16.7)	7 (22.6)	1 (1.9)	
B2B3: stricturing and/or penetrating	2 (6.7)	1 (3.2)	1 (1.9)	
P: perianal	23 (76.7)	6 (19.4)	19 (36.5)	< 0.01
Disease activity (at the diagnosis)				
PCDAI (mean ± SD)	50.9 ± 12.3	40.0 ± 11.9	35.4 ± 12.4	< 0.01
PUCAI (mean ± SD)	31.3 ± 16.5	44.0 ± 18.4	44.1 ± 20.0	0.46

### Medical treatment

The treatment of IBD followed a standardized protocol for patients according to the guidelines as described [20, 21]. Induction therapy and maintenance therapy of the first year is displayed in Table 3. Two infantile patients after colectomy and colostomy were remission with total enteral nutrition (TEN). The patients with GSD Ib were treated with granulocyte colony-stimulating factor (G-CSF) and mesalazine. Forty-two (29.4%) patients were steroid-dependent. Most patients received antibiotics as necessary during the course. Supportive treatments, including parenteral or enteral nutrition, immune globulin, albumin, transfusion with concentrated red cells, were given as necessary.

**Table 3:** Main medical treatment of CD and UC

Treatment	Number of patients (%)		
	CD (*n* = 113)	UC (*n* = 30)	IBD (*n* = 143)
Induction therapy			
Corticosteroids	57 (50.4)	18 (60.0)	75 (52.4)
Mesalazine	29 (25.7)	25 (83.3)	54 (37.8)
Biological agent	65 (45.5)	5 (16.7)	70 (49.0)
Thalidomide	14 (12.4)	3 (10.0)	17 (11.9)
Maintenance therapy^a^			
Azathioprine	54 (47.8)	6 (20.0)	60 (42.0)
Biological agent	48 (42.5)	2 (6.7)	50 (35.0)
Thalidomide	32 (28.3)	3 (10.0)	35 (24.5)
Mesalazine	23 (20.4)	21 (70.0)	44 (30.8)
Methotrexate	3 (2.7)	0	3 (2.1)
Cyclosporine A	2 (1.8)	0 (0.0)	2 (1.4)
Total enteral nutrition	2 (1.8)	0 (0.0)	2 (1.4)
Corticosteroids	31 (27.4)	11 (36.7)	42 (29.4)

^a^The data are from an analysis of the first year of maintenance therapy

### Surgical treatment

**Fig. 1:**
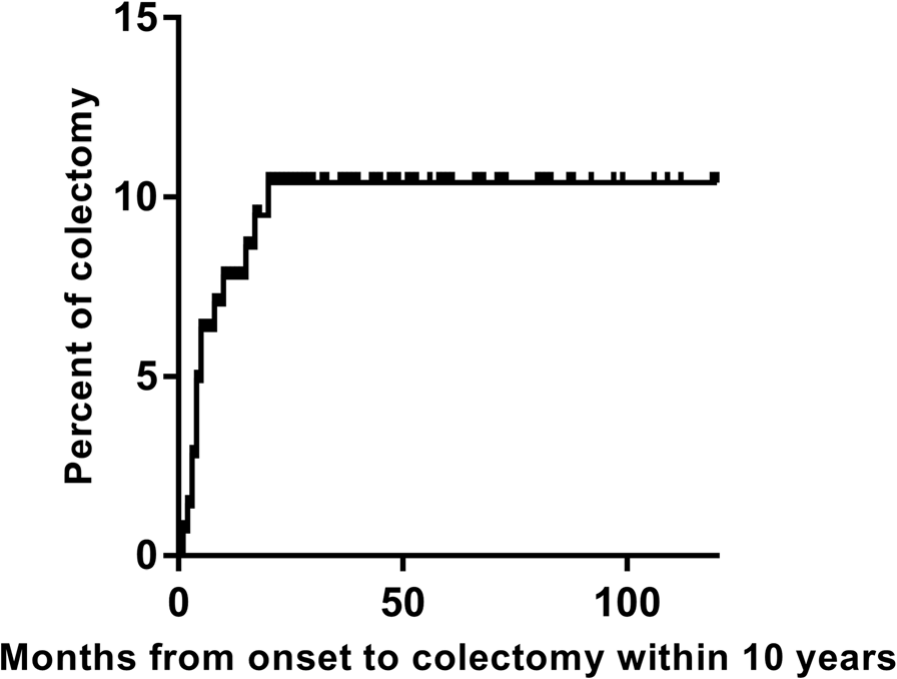
Kaplan-Meier curve of time from onset to colectomy within 10 years follow-up

There were 15 (10.5%) patients who underwent abdominal surgeries, including 14 CD patients and 1 UC patient. The surgeries were carried out in the median duration of 5 months after disease onset; 14/15 patients underwent surgery within 20 months, and the remaining patient had surgery at 190 months. Figure 1 shows the Kaplan–Meier curve of time from onset to colectomy within 10 years. There were three major indications: confirmed diagnosis with exploratory laparotomy (3/15, 20.0%), intestinal perforation surgery (7/15, 46.7%) and aggressive disease after medical treatment (5/15, 33.3%). Cox univariate analysis showed that surgery was only associated with B3 behaviour (HR: 10.2; 95% CI: 3.35–31.36; *P* < 0.01). Intestinal or ileocaecal segment resection was performed in all patients; 9/15 patients underwent colostomy or ileostomy simultaneously. Up to the latest follow-up, one patient with perforation died after surgery; one patient who underwent re-anastomosis of the bowel 5 months after colostomy died of sepsis after relapse. One patient had persistent disease activity after reconnection of the bowels. Six patients went into remission after colostomy, including two patients with TEN and others with medical therapy. The others were managed with medical treatment after surgery.

### Death analysis

**Fig. 2:**
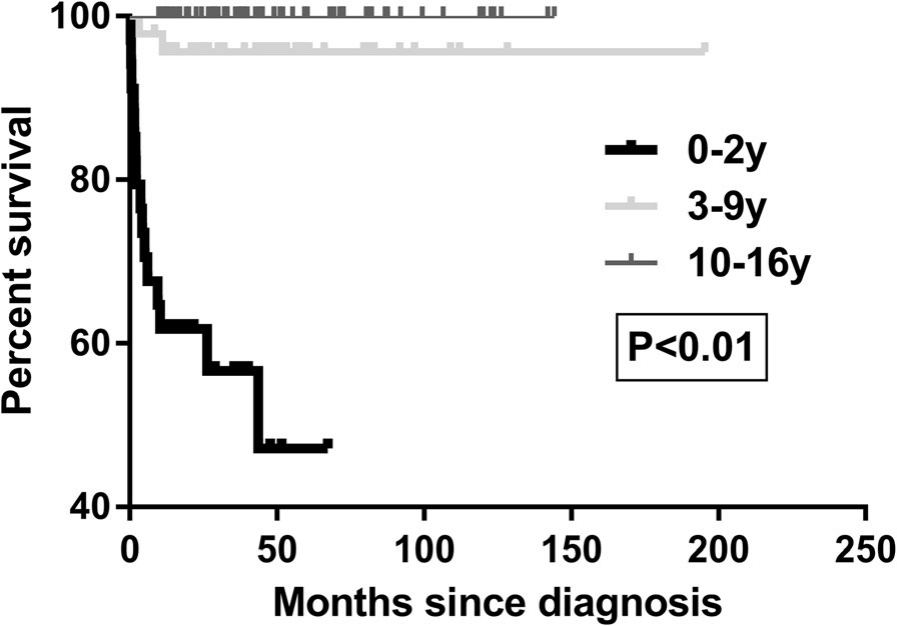
Kaplan-Meier curves showing time from diagnosis to death. Log rank test for equality of survival curves, *P <* 0.01

Seventeen patients (11.9%) died, all of whom were CD patients. The characteristics of these patients are listed in Table 4. The median diagnosis age was 1 year old (range, 0.16–5 years old). The median time from diagnosis to death was 3.7 months (range, 0.4–43.7 months). Fourteen patients died of various complications of CD, the majority from serious infections such as sepsis, while one patient died of sepsis after UCBT. Three died of intestinal perforation with or without surgery. Cox univariate analysis showed that the death of CD patients was associated with age, perianal disease, B3 behaviour and z-value of weight < − 2 (*P* < 0.01). Further multivariate analysis clearly suggested that age 0–2 y and B3 behaviour were risk factors for death (Table 5). Figure 2 shows the cumulative survival rates of various age groups. There was no difference in mortality between patients with or without gene mutations in the 0–2 y group.

**Table 4:** Characteristics and clinical manifestations of patients who died

No.	Genetic Test	Age of diagnosis (y)	Initial presentation	Phenotype	Family history	Medication	Surgery	Cause of death	Months from diagnosis to death	Year of death
1	NA	3	Abdominal pain, fever	B3 + P	None	CS	Colectomy and enterostomy	Intestinal perforation	3.2	2007
2	NA	2.3	Diarrhoea, Blood in stool	B2	None	CS	–	Infection	1.5	2007
3	NA	5	Diarrhoea	B3 + P	None	CS, MES	–	Infection	11.2	2009
4	NA	0.6	Diarrhoea	B1 + P	None	IFX, CS	–	Infection	4.2	2010
5	NA	2	Diarrhoea	B2 + P	A brother died of diarrhoea	IFX, CS	Enterostomy	Infection	5.0	2011
6	NA	1	Diarrhoea	B2B3 + P, L4a	None	CS	–	Infection	0.6	2011
7	NA	1.5	Diarrhoea, fever	B2 + P	None	CS, AZA	–	Infection	43.7	2015
8	NA	0.3	Diarrhoea, fever	B3 + P	None	CS	–	Infection	2.0	2012
9	ND	1.5	Diarrhoea, fever	B2 + P	None	IFX, CS, THD	–	Intestinal perforation	26.3	2014
10	IL-10RA	0.83	Diarrhoea, fever	B1 + P	None	IFX, CS, THD	–	Infection	6.1	2014
11	NA	0.83	Diarrhoea, fever	B2B3 + P, L4b	None	CS	–	Intestinal perforation	0.5	2014
12	NA	0.16	Diarrhoea, fever	B3 + P	None	CS	–	Infection	2.1	2015
13	ND	2.5	Diarrhoea, fever	B2 + P	None	IFX, CS	–	Infection	3.7	2014
14	IL-10RA	0.8	Diarrhoea, fever	B3 + P	No.17’s sister	THD	–	Infection	1.6	2014
15	ND	1	Diarrhoea, fever	B2 + P	None	CS	–	Infection	10.4	2016
16	IL-10RA	1.67	Diarrhoea, fever	B1 + P, L4a	None	Antibiotics only^a^	–	Infection	0.4	2016
17	IL-10RA	0.58	Diarrhoea, fever	B1	No 14’s younger brother	UCBT	–	Infection after UCBT	9.5	2016

*AZA* azathioprine, *CS* corticosteroids, *EN* enteral nutrition, *IFX* infliximab, *MES* mesalazine, *NA* not available, *THD* thalidomide, *ND* not detected, *UCBT* umbilical cord blood trans-plantation

^a^Other patients may also use antibiotics but not list in the table

**Table 5:** Univariable and multivariable analyses of clinical variables influencing death in CD

Variables	Univariable analysis			Multivariable analysis (significant in univariate)		
	HR	95% CI	*P* value	HR	95% CI	*P* value
Age						
3–9 y vs 0–2 y	0.09	0.02–0.38	< 0.01	0.11	0.18–0.61	0.01
10–16 y vs 0–2 y	0.01	0.00–0.00	0.93	0	0–0	0.92
Sex						
female vs male	0.35	0.10–1.22	0.10			
Disease location						
L2 vs L1	7.15	0.92–55.37	0.06			
L3 vs L1	1.40	0.16–11.94	0.76			
L4 vs no L4	0.48	0.14–1.68	0.25			
Perianal disease vs not	12.37	2.81–54.80	< 0.01	3.45	0.69–17.28	0.13
Behaviour B2 vs not	1.02	0.39–2.64	0.97			
Behaviour B3 vs not	5.23	1.98–13.79	< 0.01	4.83	1.57–14.87	< 0.01
Weight < -2SD vs not	5.96	2.20–16.20	< 0.01	1.51	0.51–4.52	0.46
Growth impairment vs not	2.01	0.77–5.29	0.16			
Top-down treatment vs not	0.40	0.09–1.74	0.22			
Steroid dependency vs not	1.75	0.67–4.60	0.26

### Relationship between sustained remission and phenotype of the disease

**Fig. 3:**
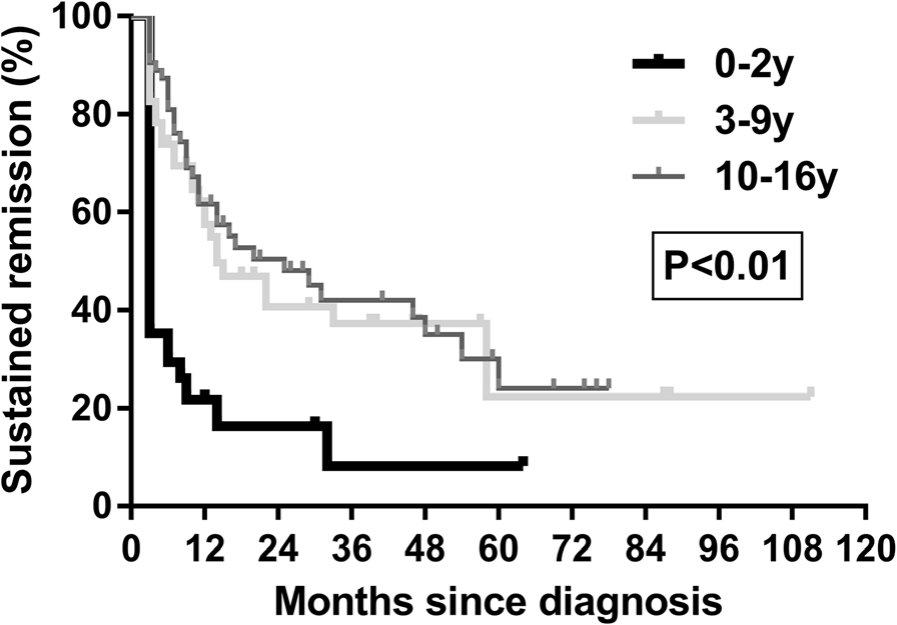
Kaplan-Meier curves showing time from diagnosis to relapse. Log rank test for equality of survival curves, *P <* 0.01

Based on follow-up data, there were 52 (36.4%) patients achieving sustained remission. There were 34 (23.8%) patients with persistent index activity after the first three-month treatment. Fifty-seven (39.8%) patients relapsed, and 27 (47.6%) of these relapsed within 1 year of diagnosis. The cumulative sustained remission rates are shown in Fig. 3. The remission varied amongst the age groups. Cox univariate analysis indicated that the relapse of CD was associated with age, perianal disease, B3 behaviour, z-value of weight < − 2 and steroid dependency. Further multivariate analysis suggested that age group 0–2 y, B3 behaviour and steroid dependency were risk factors (Table 6). There was no difference of remission between patients with or without gene mutations in the 0–2 y group.

**Table 6:** Univariable and multivariable analyses of clinical variables influencing relapse in CD

Variables	Univariable analysis			Multivariable analysis (significant in univariate)		
	HR	95% CI	*P* value	HR	95% CI	*P* value
Age						
3–9 y vs 0–2 y	0.25	0.14–0.48	< 0.01	0.26	0.13–0.53	< 0.01
10–16 y vs 0–2 y	0.20	0.11–0.36	< 0.01	0.23	0.12–0.44	< 0.01
Sex						
female vs male	0.84	0.52–1.36	0.47			
Disease location						
L2 vs L1	2.18	0.98–4.84	0.06			
L3 vs L1	1.29	0.61–2.77	0.51			
L4 vs no L4	0.89	0.54–1.46	0.64			
Perianal disease, yes vs no	1.59	1.00–2.53	0.048	1.13	0.68–1.88	0.63
Behaviour B2 vs not	1.04	0.66–1.64	0.87			
Behaviour B3 vs not	2.08	1.15–3.77	0.02	1.96	1.01–3.81	0.046
Weight < -2SD vs not	1.71	1.04–2.82	0.04	0.96	0.54–1.69	0.88
Growth impairment vs not	1.31	0. 83–2.09	0.25			
Top-down treatment vs not	0.69	0.40–1.21	0.20			
Steroid dependency vs not	2.26	1.40–3.64	< 0.01	1.88	1.16–3.06	0.01

## Discussion

In this study, we analysed the relationship between disease phenotype and prognosis of paediatric inflammatory bowel disease in China. Younger patients may have extensive and aggressive disease. In the present study, 56% of paediatric IBD patients were younger than 10 years and 23.8% patients were younger than 3 years old. By contrast, the rate was quite low in a study carried out in Italy in which the VEO-IBD (0–3 y) was only 4%; however, the fundamental difference was that patients with genetic defects were excluded [22]. In addition, the proportion of patients younger than 10 years old were also higher than in other studies (23.2–50%) [22–25]. The explanation may be that, as a hospital specializing in refractory IBD, we observed more refractory patients who were recruited for this study and the patients recruited were much younger.

DNA sequencing tests for VEO-IBD patients were performed in the current study since 2012, and we found that 58.3% of the tested patients had monogenetic disorders with the gene mutation on IL-10 receptor A (IL-10RA). Glocker et al. first reported in 2009 that the mutation of IL-10 receptor caused IBD [26]; since then, IL-10 receptor defects have drawn much attention by paediatric IBD researchers worldwide [27, 28]. It is interesting that most studies in East Asia including our results pointed out the dominance of IL-10RA mutations in IBD patients. By contrast, based on the European data, the numbers of patients with IL-10RA and IL-10 receptor B (IL-10RB) mutation were somewhat equivalent. In addition, our data demonstrated that none of our patients suffered from lymphoma and no parents were consanguineous; these observations are also different from those of the European survey [28]. Because of the high percentage of positive findings, one would speculate that there should be more patients with gene mutations related to IBD in those diagnosed before 2012 who were not examined by the NGS test. Furthermore, some studies demonstrated that many monogenetic disorders may cause IBD [29]. We had a patient who developed IBD secondary to GSD Ib, something that has not been reported in China before. It is noteworthy that 77% of GSD Ib patients may have IBD, and for these patients, recommended treatment is with G-CSF and mesalazine rather than with steroids [30, 31]. We also diagnosed two younger patients with diarrhoea as having chronic granulomatous disease (CYBB mutation) and hyper-IgM syndrome (CD40LG mutation). Both of which have been reported as monogenetic disorders causing IBD. Since they did not have typical IBD endoscopic and pathological manifestations, these two patients were not included in the study. Another study also reported mutations in EPCAM, TNFAIP3 and LRBA in China. However, based on all the data, it has been confirmed that IL-10 RA may be the main mutation in China [32, 33].

The clinical manifestations and phenotypes of diseases were analysed for patients diagnosed in the current study. Patients in the 0–2 y group commonly manifested systemic symptoms such as fever, weight loss and limitation of activity, colonic lesions, strictures and perianal disease; this was similar to what was reported in other studies [34]. It was reported that patients usually had accompanying extra-intestinal symptoms of joints, skin, liver, and eyes with percentages as high as 10–20% [23, 35]; however, few patients in the current study developed these extra-intestinal symptoms. It is worth noting that there were two patients with histories of juvenile rheumatoid arthritis (JIA) in the study; further investigation is needed to elucidate whether JIA and IBD are associated with the same pathogenesis.

Our centre has a standardized protocol of treatments for IBD patients. Of the total, 76.2% went into remission or improved after induction therapy. However, patients younger than 3 years old may relapse or suffer from various complications, possibly leading to a poor prognosis. Our findings indicated that infantile patients, penetrating lesions and steroid dependency were risk factors for poor prognosis, a finding that accorded with the consensus guidelines of ECCO/ESPGHAN [20].

Death reports were relatively high in this study, and all the deaths were in patients < 6 years old with CD. Most of these were very sick when they were recruited at the centre and half of them died within the first 3 months after diagnosis. Some patients responded to the treatment of IBD but developed infections and died unexpectedly. Some deaths were confirmed as being associated with IL-10RA defects. It was reported that IL-10RA mutations affected immune function, and patients with IL-10RA mutations may have a poorer prognosis [13]. Fortunately, IL-10RA and IL-10RB mutations can now be cured through haematopoietic stem cell transplantation [9, 36].

Based on our follow-up data, patients with a poor response to medicine would improve after colectomy, especially combined with colostomy. It was reported that all patients with IL-10RA and IL-10RB mutations need surgical interventions, including partial or subtotal colectomy. This may prolong survival time, but cannot help patients achieve remission [37]. Our experience showed that colostomy might be an effective therapy that can maintain clinical remission but cannot lead to mucosal healing. As some patients refused to have colostomy when advised, the reported percentage of surgery may be lower than medically necessary. Because a limited number of patients had long follow-up of colostomy, more clinical observation is required.

Our results have some limitations. As a hospital specializing in refractory IBD, the clinical features of patients presenting with refractory IBD differ from those of the general population. This may cause referral bias. A national IBD network should be set up to recruit more patients within the Chinese population. Another limitation was that, as a retrospective study, non-standardized documentation may have resulted in the inability to determine disease prognosis with various treatments. Furthermore, antibiotics and supportive treatment including parenteral or enteral nutrition were not recorded in detail. The potential variability in treatment practices could impact outcomes. It is worthwhile to set up a randomized controlled trial to analyse the long-term efficacy and safety of these medicines in different age groups. It is worth noting that the development of diagnostic methods during our study period may have affected the evaluation and treatments in the study. For example, genetic testing may have impacted the rate of detection of monogenic disease and changes in investigations (MRE and capsule endoscopy) over the study period, possibly impacting determination of the extent of small bowel Crohn’s disease.

## Conclusions

We determined that age was the major factor determining the various clinical manifestations and prognoses for IBD patients. Infantile IBD may be caused by monogenic defects, particularly IL-10 RA mutations. Colostomy can improve clinical symptoms, but haematopoietic stem cell transplantation might cure these patients. NGS should be performed for each VEO-IBD, especially for infants. It is necessary to incorporate genetic testing into medical insurance plans and to regard it as a routine examination. This will improve the diagnosis and treatment of VEO-IBD in China.

## Abbreviations

ACMG: American College of Medical Genetics
AZA: Azathioprine
CD: Crohn’s disease
CI: Confidence interval
CS: Corticosteroids
EN: Enteral nutrition
ESR: Erythrocyte sedimentation rate
G-CSF: Granulocyte colony-stimulating factor
GI: Gastrointestinal
GSD: Glycogen storage disease
HR: Hazard ratio
IBD: Inflammatory bowel disease
IBD-U: IBD-unclassified
IFX: Infliximab
IL-10 RA: IL-10 receptor A
IL-10RB: IL-10 receptor B
IQR: Interquartile range
JIA: Juvenile rheumatoid arthritis
MDT: Multi-disciplinary team
MES: Mesalazine
MTX: Methotrexate
NA: Not available
ND: Not detected
NGS: Next generation sequencing
PCDAI: Paediatric Crohn’s Disease Activity Index
PUCAI: Paediatric Ulcerative Colitis Activity Index
SD: Standard deviation
TEN: Total enteral nutrition
THD: Thalidomide
UC: Ulcerative colitis
UCBT: Umbilical cord blood trans-plantation
VEO-IBD: Very early onset IBD
WES: Whole exome sequencing
y: Years
